# Rare Lower Extremity Fibrosarcomatous Variant of Dermatofibrosarcoma With Myxoid Features Treated With Transtibial Amputation

**DOI:** 10.7759/cureus.18079

**Published:** 2021-09-18

**Authors:** Elizabeth K Pryor, Margaret A Sinkler, Asad Ullah, Elizabeth Martin, Kelly Homlar

**Affiliations:** 1 Orthopedics, Medical College of Georgia - Augusta University, Augusta, USA; 2 Pathology, Medical College of Georgia - Augusta University, Augusta, USA; 3 Orthopedic Surgery, Medical College of Georgia - Augusta University, Augusta, USA

**Keywords:** dermatofibrosarcoma protuberans, immunohistochemistry, myxoid change, fibrosarcomatous transformation, transtibial amputation

## Abstract

Dermatofibrosarcoma protuberans (DFSP) is an uncommon soft tissue tumor originating from the dermis, with high rates of local recurrence and invasive growth but low likelihood of distant metastasis. Fibrosarcomatous transformation (FS-DFSP) of DFSP accounts for approximately 5-15% of DFSP tumors, is a higher-grade tumor, with higher chances of metastasis and poorer prognosis. We present a case of a 66-year-old female presented with a large fungating mass on the left dorsal foot. Ultrasound-guided core needle biopsy with immunohistochemistry suggested a spindle cell neoplasm, favoring myxofibrosarcoma with intermediate grade. The patient elected for below-knee amputation over limb salvage with wide resection and free flap reconstruction. Based on clinical presentation, radiologic, histologic features and fluorescence in situ hybridization (FISH) studies confirmed the diagnosis of fibrosarcomatous variant of dermatofibrosarcoma protuberans with myxoid change. FS-DFSP with myxoid change is a rare soft tissue tumor that requires aggressive treatment due to its high rates of recurrence. This case presents a rare tumor in a unique location that was successfully treated with limb amputation, which is not documented in current literature.

## Introduction

Dermatofibrosarcoma protuberans (DFSP) is an uncommon soft tissue tumor involving the dermis and underlying soft tissue with an incidence rate of 0.8-4.5 cases per million people annually [1]. DFSP usually arises between the ages of 20-40 years with no gender predilection and is characterized by aggressive local growth and high rates of local recurrence after excision, ranging from 20-50% [2, 3]. While locally aggressive, DFSP has low rates of distant metastasis and tumor-related deaths [4]. Clinically, DSFP presents as a firm plaque usually on the trunk (42-72%) but is also seen on the proximal extremities (16-30%) and head and neck (10-16%) [3, 5].

While a majority (85-90%) of DFSP are low grade, 5-15% of DFSP undergo fibrosarcomatous (FS) transformation [4]. FS-DFSP is a higher-grade tumor with higher rates of local recurrence, and they account for the majority of cases that metastasize [6, 7]. FS-DFSP can present as a primary lesion or as a recurrence of DFSP. Diagnosis of FS-DFSP is established histologically, and treatment usually involves surgical excision. We present a case of a FS-DFSP with myxoid features located on the dorsum of the left foot treated with amputation. Extremity FS-DFSP with myxoid features has been very rarely reported and treatment requiring amputation is also distinctly unusual [8, 9].

## Case presentation

**Figure 1:**
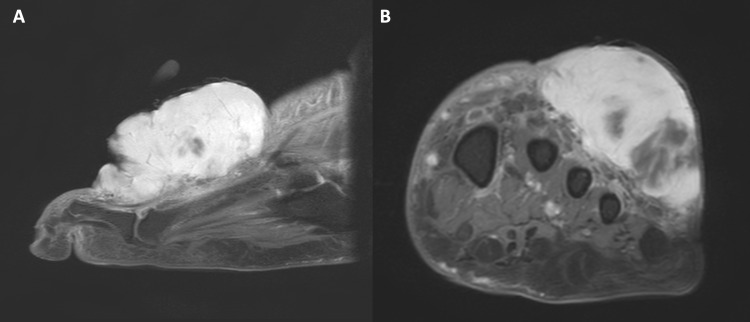
Post contrast fat-saturated T1 weighted MRI of the patient’s left forefoot with (A) sagittal and (B) axial views showing a hyperintense mass in the dorsolateral subcutaneous soft tissue measuring 3.6 x 3.9 x 6.8 cm

A 66-year-old female presented for evaluation of a 2.8 x 5.2 x 6.8 cm left dorsal foot fungating mass. She initially noticed a rash and skin irregularity on the left foot three to four years prior to presentation. The mass increased considerably in size and had begun bleeding upon minor trauma, which prompted her to seek medical attention (Figure 1).

**Figure 2:**
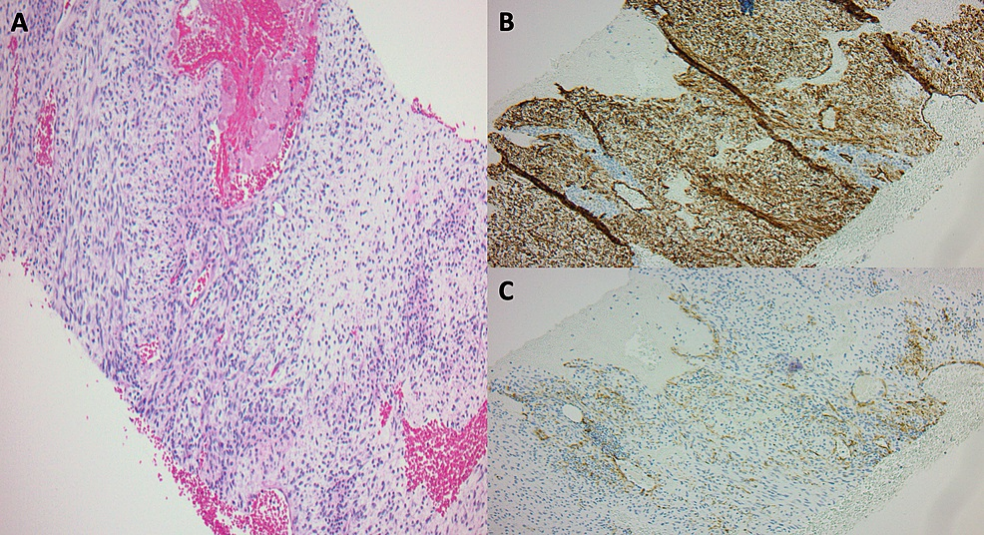
(A) Hematoxylin and eosin stain at 20x magnification showing spindle to epithelioid cells, with moderate nuclear pleomorphism and myxoid stroma. (B) CD 34 stain showing strong and diffuse staining in tumor cells. (C) SMA stain showing focal and weak staining of tumor cells. SMA: Smooth Muscle Actin

**Figure 3:**
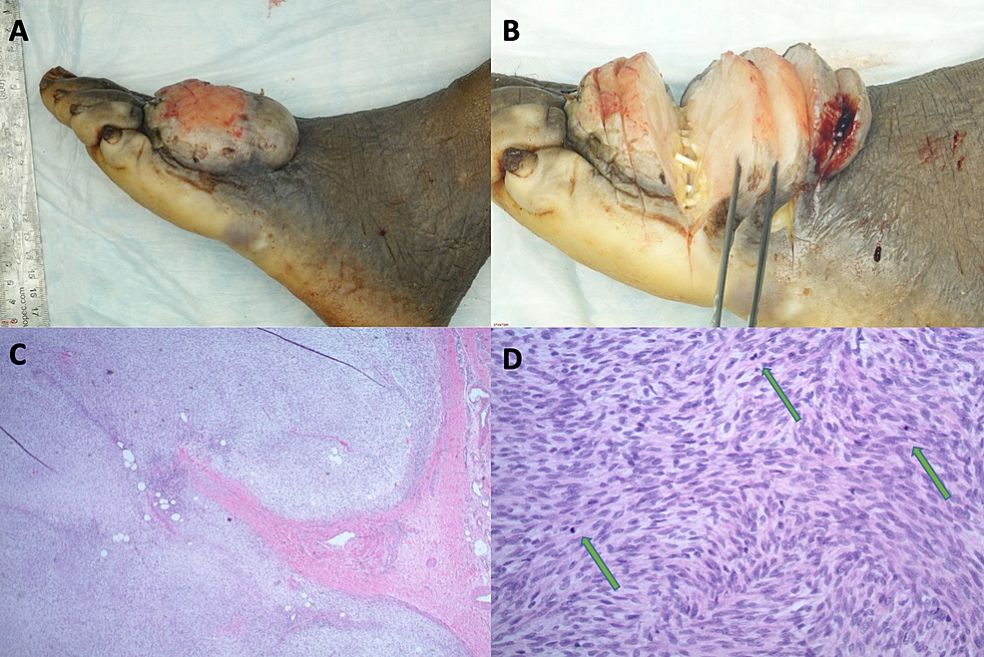
(A and B) Gross pathologic specimen demonstrating a grossly, nodular and ulcerated lesion on dorsum of the foot. (C) Hematoxylin and eosin stain at 10x magnification showing a well-circumscribed lesion entrapping fat with myxoid stroma. (D) Hematoxylin and eosin at 40x magnification showing herringbone pattern of spindle cells with multiple identifiable mitoses (arrows).

The patient underwent an ultrasound-guided core needle biopsy which revealed spindle to epithelioid cells with moderate nuclear pleomorphism, myxoid stroma, and prominent thin-walled vessels. Immunohistochemistry revealed the lesion was strong and diffusely positive for CD34, focal and weak staining with smooth muscle actin (SMA). The lesion was stained negatively for S100, epithelial membrane antigen (EMA), Desmin, and MUC-4 negative (Figure 2). Based on morphology and pattern of immunohistochemical staining, a diagnosis of “spindle cell neoplasm, favoring myxofibrosarcoma with intermediate grade” was rendered. Limb salvage with wide resection and free flap reconstruction versus below-knee amputation was discussed and the patient elected for below-knee amputation. Grossly, her amputation specimen showed a 7.5 x 5.5 x 2.8 cm nodular mass with superficial ulceration on the dorsum of the foot. Serial sections of the lesion revealed a well-circumscribed, firm tan-white lobulated cut surface with areas of myxoid change, and possible hemorrhage and necrosis. The lesion appeared to focally infiltrate the underlying soft tissue but was mobile and non-fixed to the underlying tendons and did not involve bone (Figure 3). Microscopically, the lesion revealed a well-circumscribed tumor with spindled to epithelioid morphology, eosinophilic cytoplasm, moderate nuclear atypia, and readily identifiable mitoses including atypical forms (>10 mitoses per 10 high power fields). Much of the lesion appeared fibrosarcomatous, however, several large foci showed completely myxoid stroma. The perimeter of the tumor showed infiltration of subcutaneous fat and focal involvement of the underlying deep tissues, but no extension into the tendon. In addition, it was noted that the tumor involving the dermis encased adnexal dermal structures without destroying them. These features raised consideration of DFSP in the differential and break-apart fluorescence in situ hybridization (FISH) for platelet-derived growth factor subunit B (PDGFB) rearrangement which has been identified in dermatofibrosarcoma protuberans was performed. Results showed “22q13(PDGFB sep)3'or5'loss,” confirming the diagnosis of DFSP. Based on clinical presentation, radiologic, histologic features and FISH studies, a final diagnosis of fibrosarcomatous variant of dermatofibrosarcoma protuberans was made. Subsequent inguinal lymph node biopsy revealed no metastasis. Oncologic staging for the tumor was pT2N0M0.

The patient had no complications related to her amputation. Based on recommendations from hematology-oncology, due to the negative lymph node biopsy and successful resection, the patient entered into continued surveillance with periodic imaging and no adjuvant therapies were recommended as treatment with imatinib is reserved for metastatic or recurrent cases of FS-DFSP.

## Discussion

Our case demonstrates the successful treatment of a rare fibrosarcomatous variant of DFSP, approximating about 5-15% of all DFSP tumors [5, 10, 11]. The mass usually grows as a tan-white raised lesion characterized by fascicles of spindle-shaped tumor cells, described as herringbone pattern of growth, with high cellularity and increased mitotic activity [4, 11, 12]. Fibrosarcomatous components are indistinguishable from a high-grade fibrosarcoma. This can be compared to the uniform, low-grade DFSP characterized by a storiform or cartwheel pattern [13]. The myxoid changes seen in our patient are rare in classic DFSP but seem to be more commonly found in FS-DFSP [4]. These tumors may be local or found invading local tissues, including subcutaneous tissue and skeletal muscle. While FS-DFSPs show considerable variability in presentation, consistently they demonstrate an association with higher rate of metastasis (10-15% vs. less than 1%) and poorer prognosis compared to low-grade DFSP [4, 5, 13, 14].

This case demonstrates one of these rare soft tissue tumors in an uncommon location. The majority of FS-DFSP are located on the trunk and proximal extremities, followed by distal extremities and head/neck [5]. FS-DFSP appears more commonly as the initial lesion but can appear as a recurrence [4, 13]. Mentzel et al. [4] looked at 41 cases of FS-DFSP, with six cases on upper extremities (shoulder) and four cases on the lower extremities (sole of foot, calf, thigh). Unlike the case presented above, all were treated by local excision. Also usual to this case is the notable myxoid change present in areas of this tumor. While it has been described in both DFSP and FS-DFSP, myxoid change is rare. Although myxoid features do not appear to alter the overall prognosis of the tumor, it is a diagnostic pitfall for the unwary pathologist as it is not usually considered as a feature of DFSP. The most important differential diagnosis of DFSP with myxoid change is myxofibrosarcoma, which is characterized by multilobular growth pattern, marked cytological atypia, and presence of curvilinear vessels [15]. As these features were lacking in the present tumor, myxofibrosarcoma was ruled out morphologically.

The prognosis of FS-DFSP with surgical resection with negative microscopic margins has been shown to result very good outcomes. Additionally, the likelihood of local recurrence depends on the extent of initial resection. Other factors such as mitotic index and patient age over 50 years are associated with a less favorable prognosis [11]. It has been well established that wide resection with negative microscopic margins is critical for local disease control. Local recurrence at five years is 28-33% with negative margins and 50-100% with positive margins, demonstrating the importance of complete surgical removal of the tumor [10, 11]. Mohs surgery and radiation therapy have both demonstrated improved clinical outcomes, but larger studies need to be done [16, 17]. Unresectable FS-DFSPs are classically treated with adjuvant radiation and/or targeted therapy, such as imatinib or sorafenib when appropriate [18]. Amputation can be considered if wide resection is not feasible or when limb salvage would lead to unreasonable morbidity or poor function [19]. There have been few reports of FS-DFSP with myxoid change that require amputation. Even complete surgical resection results in high rates of recurrence, therefore aggressive treatment is critical. Due to the unique location as well as large size and fungating nature of the presenting tumor, amputation was recommended.

## Conclusions

In conclusion, FS-DFSP represents a rare soft tissue tumor, diagnosed primarily by histology and treatment usually with surgical excision. Due to the aggressive nature of FS-DFSP, we highlight the importance of complete resection with negative microscopic margins to avoid local recurrence. The case presented describes a unique presentation and successful treatment of FS-DFSP which is not documented in current literature.
